# Difference between Consciousness of Intervention for Improving Lifestyle by Public Health Nurses and Recipients of a Company Health Check

**DOI:** 10.2188/jea.12.287

**Published:** 2007-11-30

**Authors:** Minoru Sugita, Yumi Otahara, Tomoko Fujishige, Kirika Sato, Akiko Tada, Mayumi Yamamoto, Yuko Takeoka, Masako Suzuki, Hisako Minowa, Yoji Hattori

**Affiliations:** 1 Department of Environmental and Occupational Health, Toho University School of Medicine.; 2 Health Care Center, NTT Data Corporation.; 3 Professional Counseling Services Corporation.

**Keywords:** consciousness of intervention, healthy lifestyle, public health nurse, recipient in health checkup

## Abstract

Improving an unhealthy lifestyle decreases risk of incidence and death of lifestyle-related diseases. Consultation about a healthy lifestyle to recipients of health checkups conducted by public health nurses is one method for such improvement. The objective in the present study was to investigate the difference between consciousness of intervention by (1) the public health nurses who conducted consultations with recipients of health checkups and (2) the recipients who were consulted by the public health nurses. Data on 1370 male white collar workers who underwent health checks were analyzed. When public health nurses determined that recipients required health consultation regarding lifestyle from the health checkup, they consulted with the recipients regarding improvement of lifestyle. The consultation regarding lifestyle included abstinence from smoking, drinking in moderation, exercise, and eating. The results of the analysis regarding difference in consciousness of the intervention by the public health nurses and the recipients show that (1) most drinkers did not think they were being discouraged to drink despite intervention by the public health nurses and (2) the smokers and the subjects with no habitual physical exercise tend to think that they were being consulted even though the public health nurses did not intervene regarding smoking and exercise.

## INTRODUCTION

Mortality, incidence, and prevalence of lifestyle-related diseases, e.g. cancer and cardiovascular diseases, are not low in developed countries^1^^)^. Accumulation of an unhealthy lifestyle from childhood involves major risk of lifestyle-related diseases^2^^-^^4^^)^. Improvement of an unhealthy lifestyle decreases the risk^5^^-^^9^^)^. Reduction of the risk is related to decrease in mortality, incidence, and prevalence rates^6^^-^^9^^)^, enhancing well-being and decreasing medical expense^10^^)^.

In order to improve an unhealthy lifestyle, it is important not only to spread information about reduction of risk of lifestyle-related diseases by improvement but also to understand the information sufficiently. One method to improve an unhealthy lifestyle is intervention through consultation regarding a healthy lifestyle to recipients in health checkups^5^^,^^9^^,^^11^^,^^12^^)^ or patients in clinics^13^^)^. Some articles^11^^,^^13^^,^^14^^)^, in which satisfaction and evaluation for the intervention regarding a healthy lifestyle by recipients have been studied, were published. The degree of understanding, satisfaction, and evaluation regarding such intervention by recipients varies^15^^)^.

A difference in recognition of evaluation regarding healthy lifestyles by medical staff and recipients has been reported^16^^)^. In the present study, the objective is to investigate difference between consciousness of the intervention by public health nurses and recipients in health checkup, e.g. are the recipients aware that they are being encouraged to abstain from smoking when public health nurses think they are intervening regarding abstinence from smoking.

## MATERIALS AND METHODS

### 1. Subjects studied

In October 1999, the health status of 1490 white collar workers of a large computer system corporation in Tokyo area was examined. The sex and age distribution is shown in Table 1. In the present study data on the 1370 male subjects were analyzed. Those of female subjects were not included because their number was not large. The mean, standard deviation, maximum, and minimum age of the male subjects were 30.67, 4.17, 21, and 39 years old, respectively.

**Table 1.** Age distribution of subjects by sex.

Age	Male	Female
21-25	112	28
26-30	525	41
31-35	498	50
36-40	235	1

Total	1,370	120

### 2. Health checkup

In the corporation a health checkup is carried out every autumn. The health checkup includes (1) a self-administered questionnaire, (2) blood, urine, and physical examinations, and (3) interview by public health nurses. The questionnaire (above 1) involves 127 questionnaire items, which are divided into items of physical symptoms, mental symptoms, occupational environment, and daily life habits. The blood, urine, and physical examinations (above 2) are general measurement of blood (e.g. serum total cholesterol concentration), urine, height, weight, and blood pressure, and chest X-rays. In the interview for health by public health nurses (above 3), they inquire each recipient in the health checkup about his health taking into account the above information obtained. When a public health nurse determines that the recipient needs health consultation regarding lifestyle, she intervenes by consulting with the recipient regarding improvement of lifestyle. The items for improvement are abstinence from smoking, drinking in moderation, exercise, and eating. An additional self-administered questionnaire survey regarding consciousness of the intervention for lifestyle by public health nurses was conducted in the health checkup of 1999.

### 3. Statistical analysis

Odds ratios were calculated using conditional and unconditional logistic regression models in the present study assigning age as a confounding variable. Age was dealt with as a dichotomous datum, i.e. from 21 to 30 or from 31 to 40 years old group. For computation SAS Release 6.12 was used.

Statistical tests for differences between two odds ratios were calculated assuming that the odds ratios were distributed normally. Summarized odds ratios, their 95% confidence intervals, and their p-values were calculated using Fleiss’s method^17^^)^.

Smoking, drinking, and exercise in the recipients’ lifestyles were dealt with as dichotomous variables. Smoking (+) means current smoking, drinking (+) excludes drinking only at banquets, and exercise (+) indicates habitual physical exercise.

## RESULTS

Tables 2-a to 2-d show the differences between consciousness of the intervention for lifestyle by the public health nurses and the recipients of the health checkup according to age group. Tables 3-a to 3-c indicate the tables by lifestyle of the recipients. The intervention items were abstinence from smoking, drinking in moderation, exercise, and eating. In Tables 3-a to 3-c the lifestyles of the recipients are smoking, drinking, and exercise. When a public health nurse determines that the recipient needs health consultation regarding lifestyle, she intervenes by consulting with the recipient regarding improvement of lifestyle. Intervention for smoking was conducted by public health nurses to some ex-smokers, who were not current smokers. Public health nurses intervened with some recipients who drank only at banquets or did habitual physical exercise regarding drinking or exercise, if they needed the health consultation. Each of the tables was made to compare (1) the number of subjects whom public health nurses thought to intervene and those who did not think they were receiving intervention by the public health nurses with (2) the number of subjects whom the public health nurses themselves did not believe they were offering intervention and those recipients who thought they were receiving intervention by the public health nurses for each item. The statistical method for indicating the difference between the two numbers of subjects is a conditional logistic analysis.

**Table 2.** Contingency tables for consciousness of intervention by public health nurses and recipients according to age group.

**2-a.** Abstinence from smoking.									

Consciousness by recipients	Consciousness by public health nurses								

	Total			21-30 years old			31-40 years old		
		
	(+)	(-)	Total	(+)	(-)	Total	(+)	(-)	Total
(+)	68	38	106	35	16	51	33	22	55
(-)	31	1,203	1,234	16	554	570	15	649	664

Total	99	1,241	1,340	51	570	621	48	671	719


**2-b.** Drinking in moderation.									

Consciousness by recipients	Consciousness by public health nurses								

	Total			21-30 years old			31-40 years old		
		
	(+)	(-)	Total	(+)	(-)	Total	(+)	(-)	Total

(+)	19	40	59	9	10	19	10	30	40
(-)	78	1,214	1,292	48	561	609	30	653	683

Total	97	1,254	1,351	57	571	628	40	683	723


**2-c.** Exercise.									

Consciousness by recipients	Consciousness by public health nurses								

	Total			21-30 years old			31-40 years old		
		
	(+)	(-)	Total	(+)	(-)	Total	(+)	(-)	Total

(+)	169	126	295	73	52	125	96	74	170
(-)	73	986	1,059	44	461	505	29	525	554

Total	242	1,112	1,354	117	513	630	125	599	724


**2-d.** Eating.									

Consciousness by recipients	Consciousness by public health nurses								

	Total			21-30 years old			31-40 years old		
		
	(+)	(-)	Total	(+)	(-)	Total	(+)	(-)	Total

(+)	181	123	304	99	48	147	82	75	157
(-)	96	952	1,048	47	435	482	49	517	566

Total	277	1,075	1,352	146	483	629	131	592	723

**Table 3.** Contingency tables for consciousness of intervention by public health nurses and recipients according to lifestyle of the recipients.

**3-a.** Abstinence from smoking.						

Consciousness by recipients	Consciousness by public health nurses					

	Recipients’ smoking (+)			Recipients’ smoking (-)		
	
	(+)	(-)	Total	(+)	(-)	Total
(+)	68	31	99	0	7	7
(-)	11	411	422	20	792	812

Total	79	442	521	20	799	819


**3-b.** Drinking in moderation.						

Consciousness by recipients	Consciousness by public health nurses					

	Recipients’ drinking (+)			Recipients’ drinking (-)		
	
	(+)	(-)	Total	(+)	(-)	Total

(+)	18	30	48	1	10	11
(-)	49	683	732	29	529	558

Total	67	713	780	30	539	569


**3-c.** Exercise.						

Consciousness by recipients	Consciousness by public health nurses					

	Recipients’ exercise (+)			Recipients’ exercise (-)		
	
	(+)	(-)	Total	(+)	(-)	Total

(+)	127	78	205	42	48	90
(-)	28	555	583	45	431	476

Total	155	633	788	87	478	566

Table 4 shows the odds ratios, their 95% confidence intervals, and p-values for statistical test for the contingency tables (Tables 2-a to 2-d) by age group using the conditional logistic analysis. The values for total subjects were obtained being adjusted by age group as a confounding variable. In the ‘Total’ column of Table 4, the only odds ratio of drinking was smaller than 1.0 with statistical significance, indicating that the number of subjects conscious of intervention for drinking by public health nurses (+) and by the subjects (-) was significantly larger than that of consciousness by public health nurses (-) and by the subjects (+). This means that the recipients were apt to think that they were not receiving intervention regarding drinking in spite of intervention by the public health nurses. In some lifestyle items, odds ratios varied pretty according to age group (Table 4).

**Table 4.** Odds retios, their 95% confidence intervals, and p-values for the contingency tables (Tables 2-a. to 2-d.) using conditional logistic analysis by age group.

	Total (age adjusted)				21-30 years old				31-40 years old			
		
	Odds R	95%-CI		p-val	Odds R	95%-CI		p-val	Odds R	95%-CI		p-val
Smoking	1.209	0.751	1.945	0.4341	1.000	0.500	2.000	1.0000	1.467	0.761	2.827	0.2527
Drinking	0.485	0.330	0.712	0.0002	0.208	0.105	0.412	0.0001	1.000	0.603	1.659	1.0000
Exercise	1.689	1.265	2.256	0.0004	1.182	0.791	1.766	0.4148	2.552	1.661	3.921	0.0001
Eating	1.263	0.966	1.651	0.0874	1.021	0.683	1.527	0.9183	1.531	1.068	2.194	0.0205

Odd R, Odds ratio; CI, confidence interval; p-val, p-value.

Tables 5-a to 5-c indicate the results derived from Tables 3-a to 3-c adjusted by age group as a confounding variable according to lifestyle of the recipients. The results in Table 4 were calculated from data on subjects with not only healthy but also unhealthy lifestyles, e.g. nonsmokers and smokers, while those in Tables 5-a to 5-c were obtained from data on smokers, drinkers, or subjects with no habitual physical exercise. In Tables 5-a to 5-c, all odds ratios for subjects with unhealthy lifestyles were statistically significant. The odds ratio of drinking was less than 1.0, while odds ratios of smoking and exercise were greater than 1.0. These results indicate that (1) most recipients did not think they were being discouraged to drink despite intervention by public health nurses and (2) the smokers and the subjects with no habitual physical exercise were apt to think that they were receiving intervention regarding smoking and exercise in spite of no intervention by the public health nurses.

**Table 5.** Odds retios, their 95% confidence intervals, and p-values for the contingency tables (Tables 3-a. to 3-c.) using conditional logistic analysis adjusted by age group according to lifestyle of recipients.

**5-a.** Abstinence from smoking.				

Recipients’ smoking	Odds R	95%-CI		p-val
(+)	2.757	1.376	5.525	0.0042
(-)	0.350	0.148	0.827	0.0167


**5-b.** Drinking in moderation.				

Recipients’ drinking	Odds R	95%-CI		p-val

(+)	0.576	0.364	0.911	0.0184
(-)	0.385	0.184	0.805	0.0112


**5-c.** Exercise.				

Recipients’ exercise	Odds R	95%-CI		p-val

(-)	2.699	1.741	4.182	0.0001
(+)	1.116	0.740	1.683	0.6000

Odd R, Odds ratio; CI, confidence interval; p-val, p-value.

In Table 6 and Tables 8 to 10, data on smokers, drinker, and subjects with no habitual physical exercise were used to obtain the results for smoking, drinking, and exercise, while the results for eating were derived from data on all subjects.

**Table 6.** p-values of tests for differences between the two odds ratios in Tables 4 and 5.

	Smoking	Drinking	Exercise	Eating
Smoking	—	0.0002	0.9593	0.0400
Drinking		—	0.0000	0.0038
Exercise			—	0.0038
Eating				—

Odds ratio	2.757	0.576	2.699	1.263

Subjects of calculating odds ratios:

Smoking: only smokers, Drinking: only drinker,

Exercise: only sabjects without habitual physical activity,

Eating: all subjects

Statistical tests for the difference between the two odds ratios in Tables 4 and 5 are shown in Table 6. Consciousness of intervention regarding drinking was remarkably different from consciousness regarding the other intervention items.

The number of subjects who thought they were receiving intervention for an intervention item in subjects whom public health nurses thought they were providing intervention for the same item should be compared with the number of subjects for the other items. A statistical comparison can be divided into (1) analysis for the difference between the numbers of subjects conscious of intervention for two items in subjects whom public health nurses thought they were intervening regarding the two same items and (2) analysis for the difference between (a) the number of subjects who thought they were receiving intervention for one intervention item in subjects whom the public health nurses thought they were offering intervention regarding the item but not for the other item and (b) the number of subjects who thought they were receiving intervention for the other item in subjects whom public health nurses thought they were intervening for the other item but not for the first item. Table 7 shows the statistical methods of comparison. In Table 7, ( I ) indicates a conditional logistic analysis [above (1)] and ( II_1_ ) vs. ( II_2_ ) indicates an unconditional (ordinary) logistic analysis {above (2)}.

**Table 7.** Statistical analyses for consciousness of intervention by recipients who accepted intervention by public health nurses (PHN).

Intervention A by PHN	Intervention B by public heslth nurses	

	(+)	(-)
(+)	( I ) Consciousness of intervention A vs. B by recipients	( II_1_ ) Consciousness of intervention A by recipients

(-)	( II_2_ ) Consciousness of intervention B by recipients	

Statistical analysis:

( I ): Conditional logistic analysis

( II_1_ ) vs. ( II_2_ ): Unconditional logistic analysis

Table 8 shows the difference between the numbers of subjects conscious of smoking and drinking in subjects whom public health nurses thought they were providing intervention for both items, which is an example of ( I ) in Table 7. Table 9 indicates the difference between (a) the number of subjects who thought they were receiving intervention for smoking in subjects whom public health nurses thought they were intervening regarding smoking but not regarding drinking and (b) the number of subjects who thought they were receiving intervention for drinking in subjects whom public health nurses thought they were intervening for drinking but not for smoking, which is the example of ( II_1_) vs. ( II_2_) in Table 7. The figures in Tables 8 and 9 were numbers of subjects with smoking (+) and drinking (+), while relationship between smoking and eating was calculated from data on smokers.

**Table 8.** Recipients’ consciousness of intervention for abstinence from smoking and drinking in moderation by public health nurses [( I ) in Table 7].

Consciousness of intervention for abstinence from smoking	Consciousness of intervention for drinking		

	(+)	(-)	Total
(+)	9	21	30
(-)	2	1	3

Total	11	22	33

**Table 9.** Recipients’ consciousness of intervention for abstinence from smoking and drinking in moderation by public health nurses [( II_1_ ) vs. ( II_2_ ) in Table 7].

Intervention by public health nurses	Recipients’ consciousness					
smoking (+), drinking (-)	smoking (+):	13	smoking (-):	3	Total:	16
smoking (-), drinking (+)	drinking (+):	1	drinking (-):	2	Total:	3

Total		14		5		19

The column of ‘Conditional logistic’ in Table 10 shows the results of statistical analysis for the difference between two intervention items for ( I ) in Table 7. The column of ‘Unconditional logistic’ in Table 10 indicates the results regarding the difference between two intervention items for ( II_1_) vs. ( II_2_) in Table 7. The column ‘Summarized odds ratio’ in Table 10 shows the summarized values of the above two odds ratios by meta-analysis^17^^)^. The values in Table 10 were adjusted by age group as a confounding variable. The age-adjusted odds ratio between smoking and drinking in Table 8 was calculated to be 10.889, which is shown in the ‘Smk,Drk’ row of Table 10, and the value calculated from data in Table 9 was 8.893, which is also shown in the same row of Table 10. These odds ratio values were greater than 1.0, indicating that recipients of the health checkup were apt to be more conscious of intervention for smoking by public health nurses than for drinking. The lowest row in Table 10 shows that the recipients were more conscious of intervention for exercise than eating.

**Table 10.** Odds ratios, their 95% confidence intervals, and p-values of association between consciousness of intervention for each pair of two items by public health nurses and recipients.

	Conditional logistic				Unconditional logistic				Summarized odds ratio			
		
	OR	95%-CI		P-val	OR	95%-CI		P-val	OR	95%-CI		P-val
Smk, Drk	10.889	2.469	48.034	0.0016	8.893	0.466	169.532	0.1462	10.452	2.777	39.347	0.0005
Smk, Exc	1.852	0.611	5.618	0.2762	0.926	0.145	5.924	0.9356	1.543	0.595	4.000	0.3718
Smk, Eat	23.785	3.111	181.887	0.0023	1.044	0.249	4.376	0.9531	4.569	0.214	97.331	0.3303
Drk, Exc	0.261	0.084	0.808	0.0198	0.026	0.002	0.354	0.0061	0.113	0.013	0.985	0.0484
Drk, Eat	0.293	0.107	0.799	0.0165	0.081	0.009	0.765	0.0282	0.232	0.088	0.613	0.0032
Exc, Eat	3.823	1.828	7.997	0.0004	1.551	0.406	5.925	0.5209	2.917	1.297	6.559	0.0096

OR, Odds ratio; CI, confidence interval; p-val, p-value; Smk, smoking; Drk, drinking; Exc, exercise; Eat, eating.

The values in Table 10 are the results for intervention (+) by public health nurses, while the results for intervention (-) can be calculated. However, those for intervention (-) could not be shown in the present study, because numbers of unhealthy subjects whom public health nurses did not think they intervened were not large enough for calculation.

In the computation of logistic analysis, the results were not influenced by exchange of independent and dependent variables. Age was dealt with a dichotomous or continuous variable as a confounding variable. The differences between the results calculated from the two types of the variables for age were not notable.

## DISCUSSION

### 1. Statistical analyses

In the present study, three kinds of statistical analyses of differences between consciousness of intervention by public health nurses and recipients in a health checkup were used. The first analysis was the statistical test in Table 6 for the difference between the two odds ratios in Tables 4 and 5 calculated from two sets of data from Tables 2-d to 3-c. For example (Tables 2-a to 3-c), the odds ratio expresses difference between (a) the number of subjects whose lifestyle public health nurses thought they were intervening and those who did not believe they were receiving intervention by the public health nurses and (b) the number of subjects whose lifestyle public health nurses did not think they were intervening and those who thought they were receiving intervention by the public health nurses regarding smoking. Comparing the number of subjects who thought they were receiving intervention and whose lifestyle public health nurses thought to intervene regarding an item with the number of subjects for the other item is the next analysis. The statistical analysis is divided into two, i.e. the second and the third analyses. The second method is conditional logistic analysis showing in ( I ) of Table 7. An example of the second analysis is the difference between the numbers of subjects aware of intervention regarding smoking and drinking and whose lifestyle public health nurses thought they were providing intervention for the two items in Table 8. The third statistical method is unconditional logistic analysis, which is shown in ( II_1_) vs. ( II_2_) of Table 7. As an example of the third analysis, Table 9 indicates difference between (a) the number of subjects who thought they were receiving intervention for smoking and whose lifestyle public health nurses thought they were providing intervention for smoking but not for drinking and (b) the number of subjects who thought they were receiving intervention for drinking and whose lifestyle public health nurses thought they were intervening for drinking but not for smoking. Table 10 shows the results of the second and the third types of analyses, which were combined by meta-analysis.

Similar comparisons were analyzed by the three statistical methods in the present study. The objects of the three analyses were different from each other. In the present study, we distinguished the three strictly. However, the results by the three methods in the present study did not differ notably from each other. It may be necessary to distinguish the three methods of analyses in some studies.

### 2. Difference between awareness of public health nurses and recipients of health checkups

In the present study, the main points of the results for the difference between awareness of intervention by the public health nurses and the recipients indicated that (1) most drinkers did not believe they were receiving intervention regarding drinking even though the public health nurses believed they were intervening and (2) the smokers and the subjects with no habitual physical exercise were apt to believe that they were receiving intervention regarding smoking and exercise even though the public health nurses did not intervene. The meaning of the above should be discussed.

Most drinkers do not think they are receiving intervention regarding drinking in spite of intervention by public health nurses. This may derive from the fact that the drinking culture is widespread in Japanese society and from awareness of studies indicating that moderate drinking is protective from lifestyle-related diseases^7^^,^^18^^)^. In spite of intervention by public health nurses, drinking may be regarded as an inevitable habit by the recipients of health checkups, and intervention regarding drinking may not leave an impression on them.

The smokers and the subjects with no habitual physical exercise were apt to think that they were receiving intervention regarding smoking and exercise in spite of no intervention by the public health nurses. This may be because smoking is well-known to be a bad habit for health, e.g. a major risk factor of lung cancer, and habitual physical exercise is recognized to be effective to reduce risk of lifestyle-related diseases^5^^,^^9^^)^.

The results of intervention for improving lifestyle by the public health nurses for the subjects with healthy lifestyles are not shown in the present study. Intervention regarding healthy lifestyle for unhealthy subjects is significant. Subjects with unhealthy lifestyles mean smokers, drinkers, or subjects with no habitual physical exercise in the present study. Analysis for eating was conducted using data on all subjects. Because habits for eating can not be easily distinguished to be healthy or unhealthy by using a simple indicator, while information on a smoking habit can be easily obtained.

A study on the differences between subjects whose awareness of intervention regarding a healthy lifestyle is concordant with awareness of public health nurses and subjects whose awareness is not should be conducted in the future. The differences should involve backgrounds, work loads, lifestyle, complaints, clinical laboratory test results, and improvement of unhealthy lifestyles.
